# Evaluation and Influencing Factor Analysis of Sustainable Green Transformation Efficiency of Resource-Based Cities in Western China in the Post-COVID-19 Era

**DOI:** 10.3389/fpubh.2022.832904

**Published:** 2022-03-21

**Authors:** Jin He Zhu, Shan Shan Wang

**Affiliations:** College of Economics and Management, Shihezi University, Shihezi, China

**Keywords:** green transformation efficiency, resource-based cities in Western China, COVID-19, Data Envelopment Analysis, Tobit model, GMM method

## Abstract

Under the impact of the coronavirus disease 2019 (COVID-19), green, low-carbon, and sustainable development has become a global consensus, and the world will enter a low-carbon and intelligent production mode faster. As the largest contributor to world economic growth and an active participant in global environmental governance, achieving green recovery and the high-quality economic and social development of China is of great significance to promote the global sustainable development strategy. The green transformation of resource-based cities in Western China is the key factor for China to build a high-quality modern economic system and promote long-term sustainable development. This article used the Super Efficiency Slack Based Model (Super-SBM) model and Malmquist index model of the Data Envelope Analysis (DEA) method to measure the static and dynamic green transformation efficiency of resource-based cities in Western China. It investigated the impact of different factors on the static and dynamic efficiency by constructing panel Tobit and dynamic panel models. The research found that the static efficiency of the green transformation of resource-based cities in Western China is low, and the development is uneven. The dynamic efficiency of green transformation showed a fluctuating upward trend first and an accelerating upward trend later. Different factors have different effects on green transformation efficiency. This article holds that the combination of post-epidemic economic recovery and green transformation is expected to promote the green transformation of western resource-based cities while injecting new vitality into China's green sustainable development in the post-COVID-19 era.

## Introduction

In 2018, at the National Conference of Ecological and Environmental Protection in China, General Secretary Xi Jinping pointed out that green development is necessary for building a high-quality modern economic system and a fundamental solution for solving pollution problems. Therefore, green development policy should be comprehensively implemented in the future. It can be seen that green transformation is the only way for China's society and economy to move toward high-quality and sustainable development. In recent years, with the advancement of the Green Belt and Road Initiative, sustainable development continues to rise in the western region. As essential strategic support for national energy resources, resource-based cities in Western China have made significant contributions to China's economic and social development. In 2020, the sudden outbreak of the coronavirus disease 2019 (COVID-19) swept the world. To block the spread of the virus, the world had to enter the epidemic prevention and control the situation through blockades and other measures, and the economic and social development was greatly interrupted. The traditional extensive economic growth model with high energy consumption, high pollution, and long-term negative impact of environmental degradation makes the green transformation of western resource-based cities even worse. With the pressure and challenges in choosing “clear waters and green mountains” and “mountains of gold and silver,” the green transformation and development of resource-based cities have become an important and arduous task for the western region. In this context, it is of great practical significance to comprehensively understand and evaluate the pattern of green transformation of resource-based cities in western China, deeply explore the different influencing factors on green transformation and development, accelerate the green transformation of western resource-based cities, and provide decision-making support for high-quality development.

Green transformation is a concept of social transformation and development arising from the natural capital constraint in recent years (1). Research on green transformation and development has always been the focus of scholars at home and abroad. After reviewing the literature, it is found that the relevant theories and practices of green transformation are still at the beginning stage, and there is no agreement on the definition of the concept. The green transformation is always confused with “sustainable development,” “green economy,” and “carbon emissions” (2–10). These keywords are the same in essence in spite of different concepts, and the research objects and methods have set a clear direction for the green transformation development in China. This article holds that green transformation is based on the theory of sustainable development, developing a green circular economy, transforming economy, society, and ecology into a green sustainable development model, and realizing the harmonious coexistence between man and nature.

The research and evaluation methods of green transformation in academia are mainly divided into two categories. One is to build a multi-dimensional logical framework and evaluation system of green transformation. For example, Chinese scholar Liu innovatively constructed a three-dimensional structural model of green transformation, including cause dimension, method dimension, and effect dimension, and evaluates the green transformation of resource-based cities from various angles, which has been recognized and used for reference by many scholars in the academic circle (11). Xiao et al. constructed an evaluation index system from three aspects: emission reduction ability, green improvement ability and competitiveness improvement, and comprehensively evaluate the green transformation development ability of 30 cities in China (12). Under the theoretical framework of green growth, Sun et al. constructed a three-dimensional space for the transformation and upgrading of resource-based cities, including energy conservation, value-added improvement, and environmental friendliness, to guide resource-based cities to get rid of the traditional development model (13). The other is the efficiency measurement of green transformation, which mainly includes the parametric method and non-parametric method. The parametric method mainly includes parametric linear programming (PLP), econometric method, and stochastic frontier analysis (SFA), while the non-parametric method mainly refers to the data envelope analysis (DEA) method. Because the DEA method does not need dimensionless processing of data, nor any weight assumption and strong objectivity, it has been more recognized and applied in the academic circle. For example, Qiu et al. used the Slack Based Model (SBM) model, spatial autocorrelation, and geographically weighted regression model to analyze the spatio-temporal heterogeneity and influencing factors of green development efficiency in the Xuzhou metropolitan area of China (14). Zhang et al., Shao et al., and He et al. researched green transformation from the perspective of total factor productivity by constructing the DEA model (15–17).

As green transformation is a hot topic of the academic circle in recent years, the relevant research conclusions are very rich. Most scholars choose one or two entry points to investigate the green transformation and development of the region, such as the Porter Hypothesis, financial development and environmental regulation (ER), carbon peak and carbon neutrality, and so on (18–23). However, through careful analysis, it can be found that the influence of the same factor may have different effects in different regions, and there are obvious spatial differences between regions. Yue et al. calculated the green development efficiency of 97 cities in China by using the SBM directional distance function, focusing on the direct impact of industrial clusters on urban green development efficiency (24). Gao et al. adopted the SBM-DEA model to measure the green innovation efficiency of China's high-tech industry considering environmental factors and studying the impact of financial innovation on green innovation efficiency (25). Zhang et al. explored the impact of ERs on the efficiency of green technology innovation in the construction industry based on the EMB model and the analysis of the evolution of Green Technology Innovation Efficiency (GTIE) (26).

Through the review and analysis of relevant literature, it can be found that the academic research on green transformation and development has never been interrupted and achieved a lot. On the whole, although the research direction and focus of scholars in various countries are different, the ultimate purpose is to achieve the optimization of resource utilization and promote the sustainable development of society. Existing research results provide great reference and guidance for this article, but we still found the following limitations after analysis: firstly, when investigating the green transformation of resource-based cities in China, the focus is mostly on resource-based cities in a certain province or a specific province as the research object, and there are only a few research on the green transformation and development of resource-based cities in the western region; secondly, there are few evaluations on the efficiency characteristics of the green transformation of resource-based cities, and most of the existing documents only measure one of the annual efficiency or the inter-period efficiency, and the comprehensiveness of the efficiency measurement is insufficient.

Therefore, based on the existing literature, this article attempts to enrich the existing research from the following two aspects: first, 38 resource-based cities in western China were selected for green transformation evaluation to enrich the regional research system of resource-based cities' green transformation in China. Second, the efficiency characteristics were used to evaluate the green transformation, and the efficiency was further divided into static efficiency and dynamic efficiency, so as to more comprehensively investigate the green transformation achievements of resource-based cities in western China. By constructing the evaluation index system of green transformation efficiency, two models under the DEA method were used to measure the two efficiencies of green transformation of resource-based cities in western China, and use the panel Tobit model and dynamic panel model to make a regression analysis on the influencing factors on green transformation efficiency. Combined with the empirical results, this article can provide a reference for formulating a more targeted and illuminating green recovery plan and realizing high-quality and sustainable development as soon as possible in the western region and even the whole country in the post-epidemic era.

## Research Object, Method, and Data Description

### Research Object

In the National Sustainable Development Plan for Resource-Based Cities (2013–2020) issued by the State Council of China in November 2013, 262 cities were classified as resource-based cities nationwide. There are 102 resource-based cities in the western region, including 48 prefecture-level cities (including prefecture-level cities, regions, autonomous prefectures, and leagues). Based on research practice and data availability, this article finally selected 38 prefecture-level resource-based cities as the research object: Baotou, Wuhai, Chifeng, Ordos, Hulunbuir, Baise, Hezhou, Hechi, Zigong, Panzhihua, Luzhou, Guangyuan, Nanchong, Guang'an, Dazhou, Ya'an, Liupanshui, Anshun, Qujing, Baoshan Zhaotong, Lijiang, Lincang, Tongchuan, Baoji, Xianyang, Weinan, Yan'an, Yulin, Jinchang, Baiyin, Wuwei, Zhangye, Pingliang, Qingyang, Longnan, Shizuishan, and Karamay.

### DEA Method and Index Selection

The Data Envelopment Analysis (DEA) is a non-parametric technical efficiency analysis method that compares evaluated objects (27). It uses convex analysis and linear programming as tools to evaluate the relative efficiency level between multi-input and multi-output Decision-Making Units (DMU).

The DEA method was first proposed by the famous American operational research scientists Charnes, Cooper, and Rhodes in 1978 (28). Because this method is purely technical and unrelated to market price, the dimensionless method is not needed to process the data. This method can directly make efficiency analysis without defining a particular functional form. It avoids the problem that it is difficult to estimate the market price due to changing factors, such as environment and resources, and the calculation is convenient and simple. At the same time, without any weight hypothesis, the optimal weight is obtained from the actual data of DMU input and output, which eliminates a large number of subjective factors and has strong objectivity. Therefore, it has been widely recognized and applied in various fields. This article used the Super Efficiency SBM (super-SBM) model and the Malmquist index (MI) model based on the DEA model to measure the static and dynamic green transformation efficiency of resource-based cities in western China to comprehensively reflect their green transformation development.

#### A Super-SBM Model Based on Undesirable Outputs

Suppose the number of evaluated DMUs is n, denoted as DM*U*_*j*_ *(j* = *1,2, …, n)*; each DMU has *m* inputs, denoted as *x*_*i*_ *(i* = *1,2, …, m)*; there are *r*_1_ kinds of desirable outputs, denoted as *y*_*s*_ *(s* = *1,2, …, r*_1_*)*; there are *r*_2_ kinds of undesirable outputs, denoted as *y*_*q*_ *(q* = *1,2, …, r*_2_*)*; *x, y*^*d*^, *y*^*u*^ represent the elements in the corresponding input matrix, desirable output matrix, and undesired output matrix; the current DMU to be measured is denoted as *DMU*_*k*_, then the model is obtained:


(1) $$\begin{array}{l} \min\theta =\frac{\frac{1}{m}\sum_{i=1}^{m}\frac{\bar{x}}{x_{ik}}}{\frac{1}{r_{1}+r_{2}}\left(\sum_{s=1}^{r_{1}}\frac{\bar{y^{d}}}{y_{sk}^{d}}+\sum_{q=1}^{r_{2}}\frac{\bar{y^{u}}}{y_{qk}^{u}}\right)}\\ s.t.\{\begin{array}{c} \bar{x}\geq \sum_{j=1,j\neq k}^{n}x_{ij}\lambda_{j}\\ \bar{y^{d}}\leq \sum_{j=1,j\neq k}^{n}y_{sj}^{d}\lambda_{j}\\ \bar{y^{u}}\geq \sum_{j=1,j\neq k}^{n}y_{qj}^{u}\lambda_{j}\\ \bar{x}\geq x_{k};\bar{y^{d}}\leq y_{k}^{d};\bar{y^{u}}\geq y_{k}^{u}\\ \lambda_{j}\geq 0 \end{array} \end{array}$$

In Formula (1), θ represents the comprehensive technical efficiency (TE) under constant return to scale (CRS), which refers to the static efficiency of the green transformation of resource-based cities in Western China. $\bar{\text{x}}$, $\bar{y^{d}}$, and $\bar{y^{u}}$ represents the optimal solutions of input and output variables in the model. λ_*j*_ is the weight vector.

#### Global Reference MI of Super-SBM Model Based on An Undesirable Output

By comparing the productivity in two periods, the Malmquist total factor productivity index analysis method can objectively reflect the changes in productivity and the effects of technical efficiency and technological progress on the changes of productivity. Therefore, MI is considered as a supplement for the DEA method. The global reference Malmquist model is an MI calculation method proposed by Pastor and Lovell in 2005: the sum of all periods of the evaluated DMU can be used as the reference set, which means that each period takes the same global frontier as reference (29). As a result, the efficiency values of each period obtained by using this model are comparable, which can intuitively demonstrate the change of productivity in each period. Then, to further observe the dynamic change of green transformation and explore the intertemporal efficiency changes, this article chose the global reference MI method based on the undesirable output super SBM model to investigate the intertemporal change of green transformation efficiency of resource-based cities in western China. To calculate the MI in different periods to evaluate the dynamic efficiency of green transformation of resource-based cities in Western China, the essential meaning of the change of the green total factor productivity is reflected through MI, to explore the development of green transformation.

Combined with Pastor's and Lovell's research results, the global reference MI period *t* to *t* +*1* under CRS is obtained [29]:


(2) $$\begin{array}{l} MI_{g}\left(x^{t+1},y^{t+1},x^{t},y^{t}\right)=\frac{E^{g}\left(x^{t+1},y^{t+1}\right)}{E^{g}\left(x^{t},y^{t}\right)} \end{array}$$

In Formula (2), *MI*_*g*_ represents the MI value, which is the dynamic efficiency value of green transformation of resource-based cities in western China.

#### Selection of Index

According to the concept of the “two mountains theory,” this article believes that the essence of measuring the green transformation efficiency of resource-based cities is to achieve the maximum economic and green output with the least factor input and ecological environment loss, and balance the “clear waters and green mountains” and “mountains of gold and silver” in the process of green transformation and development of western resource-based cities.

(1) Input indicators. Input indicators include labor, capital, energy, and land inputs. Combined with the existing research results, labor input is calculated by the total number of people employed in the whole society over the years in each city (30–32). Capital input is calculated by the investment in fixed assets of the whole society. Energy input is calculated by the electricity consumption of the whole society in each city. Land input is calculated by urban built-up area.
(2) Output indicators, including desirable output and undesirable output. Desirable output refers to the total economic output and green area of built-up area, and undesirable output refers to three environmental pollution indicators: “waste gas,” “wastewater,” and “smoke (powder) dust” (hereinafter referred to as “three wastes”). According to the existing research results, the total economic output can be measured by the gross domestic product (GDP) of each city (33–35). Then, the actual GDP output can be converted by the GDP index with 2004 as the base year, which avoids the impact of price fluctuation on calculation. The green area of the built-up area is measured by the green coverage area of the whole social built-up area of each city. “Three wastes” takes the total industrial sulfur dioxide emission, total industrial wastewater emission, and total industrial smoke (powder) dust emission of each resource-based city as the proxy variables of the three.

### Panel Regression Model and Selection of Index

In this article, the green transformation efficiency (including static efficiency and dynamic efficiency) of resource-based cities in Western China is taken as the explained variable. Based on current research results, advanced industrial structure (AIS), regional openness (RO), technological innovation (TI), ER, level of economic development (GDPP), level of urbanization (URB), and human capital (HC) are selected as explanatory variables (Table 1) (36–39). Among them, as one of the explained variables, the static efficiency value is a set of limited data with a minimum value of 0. Errors and deviations may occur if using Ordinary Least Squares (OLS) to regress the model. Therefore, the panel Tobit model is adopted in this article to estimate the relevant parameters, and the model is obtained as Formula (3):


(3) $$\begin{array}{l} TE_{it}^{*}=\alpha X'it+\varepsilon_{it}\\ \varepsilon_{it}-N\left(0,\;\sigma^{2}\right)\\ TE_{it}=\{\begin{array}{c} TE_{it}^{*}\;if\;TE_{it}^{*}>0\\ \;0\;if\;TE_{it}^{*}\leq 0 \end{array} \end{array}$$

In Formula (3), *TE*_*it*_ represents the static efficiency value, which is the actually observed explained variable. ${TE}_{it}^{*}$ is an unobservable latent variable. *X*_*it*_ represents the set of influencing factors. *i* and *t* represent region (city) and period (year), respectively. α is the regression coefficient. The random disturbance term ε_*it*_ obeys the normal distribution with mean value 0 and variance σ^2^. When the latent variable ${TE}_{it}^{*}$ is >0, *TE*_*it*_ is equal to ${TE}_{it}^{*}$ itself; when ${TE}_{it}^{*}$ is ≤0, *TE*_*it*_ is equal to 0.

**Table 1:** Variable of panel regression model.

**Variable name**	**Explanation**
TE	Calculated by Super-SBM model based on undesirable output: if TE >1, it refers to a high efficiency; if TE is equal to 1, it is valid; if TE <1, it refers to a low efficiency.
MI	Calculated by global reference Malmquist index model: if MI is >1, it means that the total factor productivity (TFP) increases; If MI is equal to 1, it means that the TFP is unchanged; if MI is <1, it means that the TFP decreases.
AIS	Output value of tertiary industry / Output value of secondary industry.
RO	Total export-import volume of the city / GDP.
TI	Financial expenditure on science and technology of the city/GDP.
ER	Calculated by entropy weight method: sulfur dioxide removal rate and industrial soot (dust) removal rate.
GDPP	Actual GDP per capita (based on 2005).
URB	Population in municipal districts at the end of the year/Total population of the whole city.
HC	Number of students in colleges and universities/Total population at the end of the year.

There may be a lag in the dynamic development process of urban green transformation efficiency in the actual social development process. Therefore, this article introduces the first-order lag term of the explained variable MI in the dynamic efficiency model to accurately obtain the dynamic panel model and regression result as Formula (4):


(4) $$\begin{array}{l} MI_{it}=\gamma +\beta_{0}MI_{it-1}+\beta_{1}X+\mu_{i}+\varepsilon_{it} \end{array}$$

In Formula (4), *MI*_*it*_ refers to the dynamic efficiency value. β is the regression coefficient. γ is the constant term. μ_*i*_ is the individual effect term. ε_*it*_ is the residual term.

### Data Source and Processing

The explained variables in this article were selected from the panel data of 38 resource-based cities in Western China from 2004 to 2016 to calculate the static value and dynamic value of urban green transformation in 12 years from 2005 to 2016. The panel data from 2005 to 2016 were taken for explanatory variables. All data come from the “*China City Statistical Yearbook*,” “*China Statistical Yearbook for Regional Economy*,” provincial statistical yearbooks, and statistical communiques of the city on national economic and social development. A few missing values were supplemented by the interpolation method. In addition, to reduce the impact of data fluctuation and heteroscedasticity on the accuracy of regression results, all variables were logarithmically processed.

## Result Analysis

### Evaluation of Green Transformation Efficiency of Resource-Based Cities in Western China

#### Static Efficiency Evaluation of Green Transformation

Based on the input-output index system and a basic model constructed above, this article used the MAXDEA 7.12.10 software (MaxDEA@maxdea.com; MaxDEA@qq.com; MaxDEA Software Ltd.) to calculate the annual static efficiency value (TE) of the green transformation of 38 resource-based cities in Western China from the global reference perspective and the perspective of the maximum output of fixed input and CRS hypothesis.

Among the 456 static efficiency values, there are 81 values whose effective values are ≥1 (the efficiency values of Tongchuan in 2012 and Nanchong in 2014 are equal to 1), accounting for 17.76% of the total sample. In other words, 82.24% of the efficiency values show low efficiency. It is worth mentioning that there are 19 effective values in 2016. It indicates that within the research range, the green transformation level of resource-based cities in Western China is obviously higher this year than in other years (Table 2).

**Table 2:** Number of cities with effective static efficiency (≥1) from 2005 to 2016.

	**2005**	**2006**	**2007**	**2008**	**2009**	**2010**	**2011**	**2012**	**2013**	**2014**	**2015**	**2016**	**Total**
Number of cities	6	8	7	6	5	5	3	8	4	5	5	19	81

**Figure 1:**
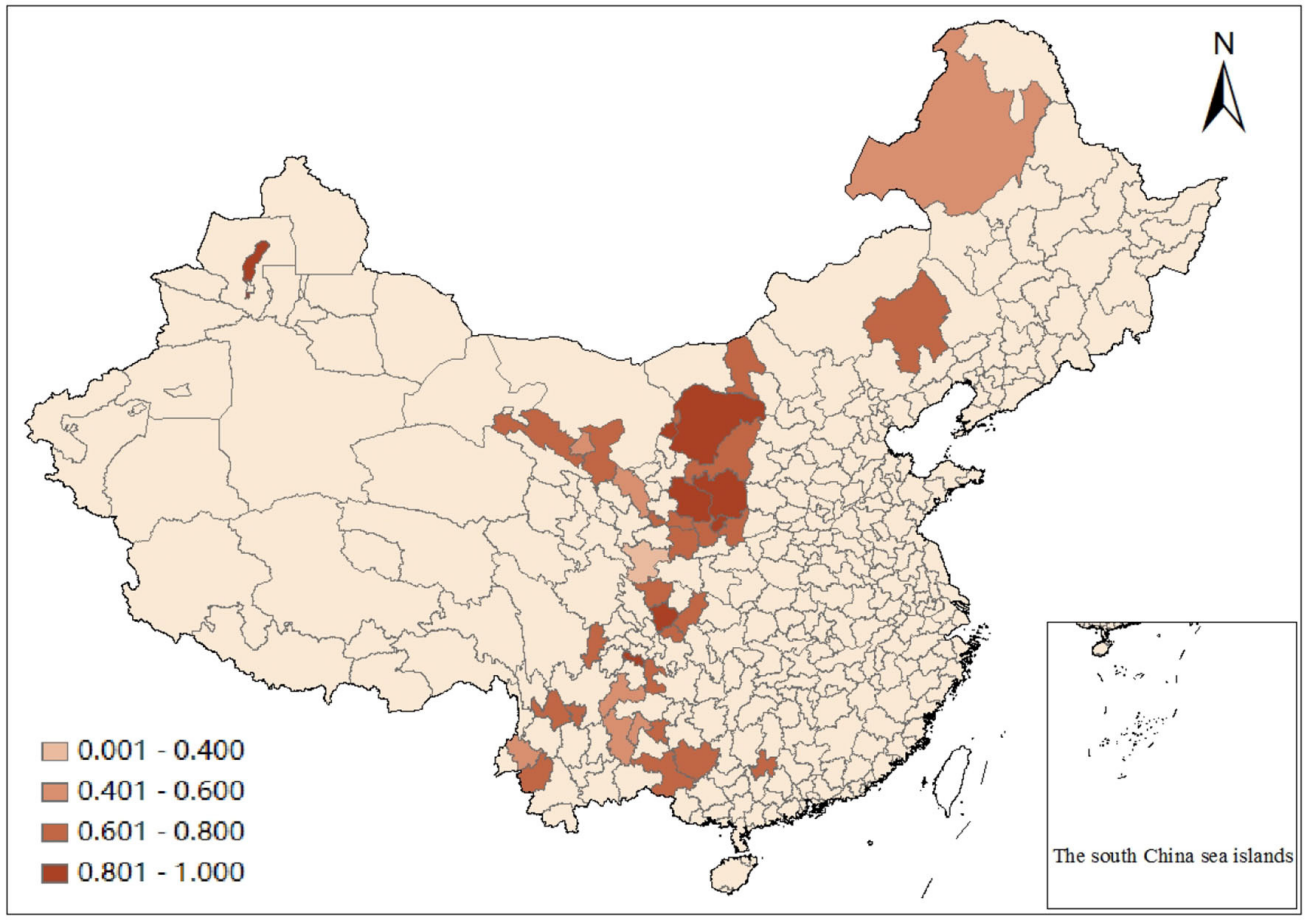
Mean static efficiency of green transformation of resource-based cities in western China from 2005 to 2016.

To understand the basic situation of the static efficiency of green transformation in each city, the annual average efficiency of 38 resource-based cities from 2005 to 2016 is further calculated. The results show that the mean values of static efficiency are all <1, with the maximum value being 0.983 in Tongchuan and the minimum value being 0.379 in Longnan. This indicates that all cities have a certain degree of resource waste in the process of green transformation, resulting in low transformation efficiency. Figure 1 can clearly show the static efficiency level of each resource-based city in Western China.

To sum up, the static efficiency of the green transformation of western resource-based cities is generally at a low level, and it is in the low-efficiency stage for most of the time.

#### Dynamic Efficiency Evaluation of Green Transformation

Based on the above efficiency evaluation indicators and basic software settings, the MI for 12 years of 38 resource-based cities in western China from 2005 to 2016 is calculated, referring to the dynamic efficiency value of green transformation.

Among the 456 dynamic efficiency values, 252 values are ≥1, accounting for 55.26% of all samples. In other words, more than half of the samples increase in MI, indicating that the efficiency of green transformation is improved compared with the previous year, and the effect of green transformation becomes better. If an MI value >1 is evaluated as excellent green transformation, then cities in 2016 have the highest excellence rate and the largest number of cities with positive dynamic efficiency growth (including 35 cities). As shown in Table 3.

**Table 3:** Number of cities with dynamic efficiency growth (> 1) from 2005 to 2016.

	**2005**	**2006**	**2007**	**2008**	**2009**	**2010**	**2011**	**2012**	**2013**	**2014**	**2015**	**2016**	**Total**
Number of cities	11	19	19	23	18	23	13	25	23	21	24	35	252

**Figure 2:**
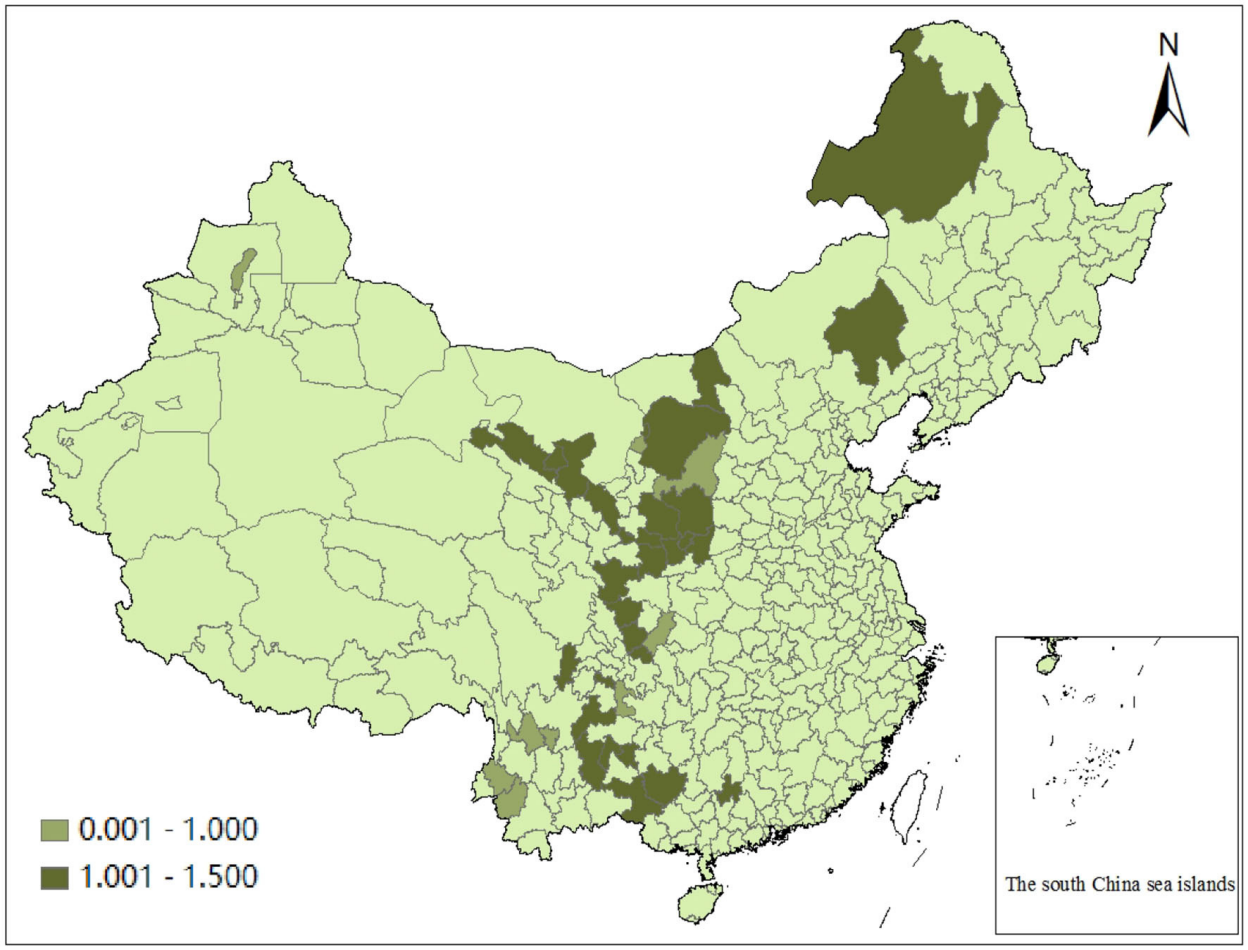
Mean dynamic efficiency of green transformation of resource-based cities in western China from 2005 to 2016.

The geometric mean is further used to calculate the average MI of each city from 2005 to 2016. According to the calculation results, there is little difference in the mean value of dynamic efficiency among cities from 2005 to 2016, and the difference between the maximum value of 1.062 (Baotou City) and the minimum value of 0.963 (Yulin City) is only 0.099, referring to that there is no obvious difference in the dynamic effect of green transformation among cities in the study area. Among them, there are 28 cities with an average value >1 and 10 cities with an average value <1, accounting for 73.68 and 26.32% of the 38 cities, respectively. It can be explained that in the 12 years from 2005 to 2016, the average dynamic efficiency of 73.68% of the resource-based cities in Western China has increased. The green transformation effect of these cities is generally desirable, and each city has made progress. The green transformation effect of the remaining 26.32% of resource-based cities is backward in a small range (Figure 2 for details).

**Figure 3:**
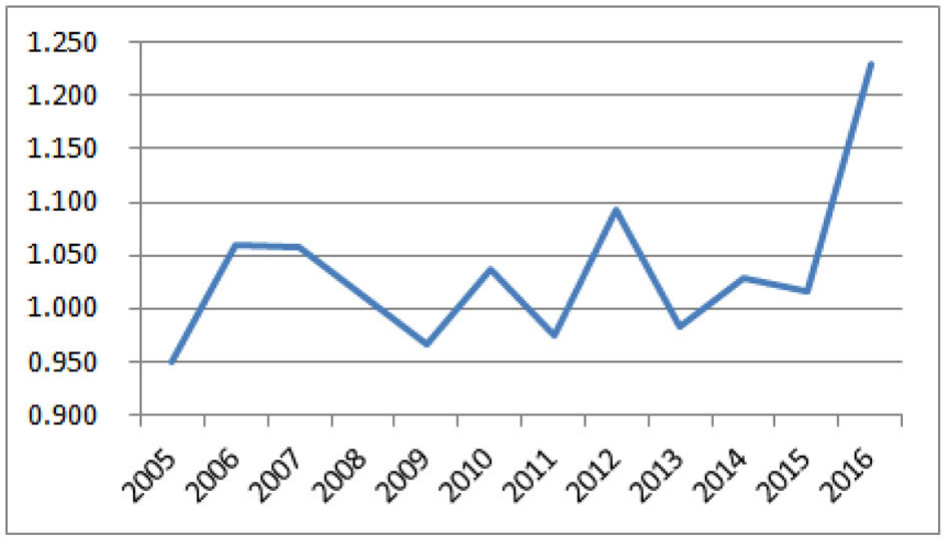
Dynamic efficiency trend of green transformation in western resource-based cities from 2005 to 2016.

As shown in Figure 3, the dynamic efficiency of the overall green transformation of western resource-based cities can be observed from a time-series perspective. From 2005 to 2015, the average dynamic efficiency slightly fluctuated, with a difference of only 0.141 between the highest year 2012 (1.092) and the lowest year 2005 (0.951). In 2016, the average value of dynamic efficiency increased significantly, reaching a peak of 1.230. The gap between this value and the lowest value in 2005 increases to 0.279. In this year, the average value of dynamic efficiency achieves the annual maximum sequential growth of 21.06%. Except that the dynamic efficiency values in 2005, 2009, 2011, and 2013 are slightly <1, the dynamic efficiency values in other years are all >1, indicating that the green total factor productivity has either high or low improvement in most years.

### Influencing Factors

#### Influence of Different Factors on Static Efficiency of Green Transformation

Regarding the Tobit model regression of panel data and based on the research of scholars, it is difficult to obtain consistent and unbiased estimators by using the fixed effect Tobit model to estimate the panel data (40). Therefore, the mixed Tobit regression model or the panel Tobit model with random effects are adopted in regression and the selection of two methods is determined by the Likelihood Ratio test (LR test). The results of the LR test for model 1 show that the original hypothesis (*P* = 0.000) is rejected, that is, the model is considered to have individual effects. For this reason, the panel Tobit model with random effects is selected for regression. The results are shown in Table 4.

**Table 4:** Regression results of influencing factors of static efficiency and dynamic efficiency of green transformation in western resource-based cities.

**Explanatory variable**	**Model 1 (Super-SBM)**	**Model 2 (Malmquist index)**
AIS	0.0286 (0.0210)	0.0281^*^ (0.0156)
RO	−0.0151 (0.0124)	−0.0047 (0.0040)
TI	0.0318^**^ (0.0156)	0.0237^**^ (0.0093)
RE	0.0183 (0.0161)	0.0347^***^ (0.0065)
GDPP	0.1278^***^ (0.0323)	0.1460^***^ (0.0110)
URB	0.0409 (0.0348)	0.0220^**^ (0.0105)
HC	−0.0991^***^ (0.0285)	−0.0002 (0.0135)
Constant	0.7435^***^ (0.2234)	−0.3344^***^ (0.1142)
LR	231.21	
*P*-value	0.000	
First-order lag term of dynamic efficiency MI		0.1445^***^ (0.0082)
AR(1)		0.0015
AR(2)		0.8853
Sargan test		1.0000

***, ** and * *indicate significance at the level of 1, 5, and 10%, respectively; the values in brackets are robust standard errors*.

It can be seen from the regression results given in the above table: first, TI significantly promotes the static efficiency of green transformation of western resource-based cities at the level of 5%. The higher the government financial investment in science and technology, the higher the static green transformation efficiency. The possible reason is that under the new situation of technological globalization and ecological environmental protection, more resource-based cities in Western China gradually abandoned the extensive resource development and management mode, and choose to adopt more advanced technology and equipment. At the same time, enterprises are strongly encouraged to improve their market competitiveness through TI. The regression results just confirm the correctness of promoting green transformation through TI. Gates Foundation CEO Mark Sussman mentioned in the latest annual open letter of the Gates Foundation in 2022 that COVID-19 has brought heavy losses to society, but also brought technological progress. Particularly in the areas of health and agriculture, we can use the research and development of new drugs to tackle major diseases, such as COVID-19, among the poor, including resource-based cities in Western China, and use agricultural innovation and transformation technologies to help cities solve problems, such as drought, to cope with the threat of climate change. It can be seen that in the post epidemic era, the static efficiency of green transformation of resource-based cities in Western China can still be improved through effective TI.

Second, the GDPP has a positive influence on the static efficiency of green transformation, indicating that the green transformation of resource-based cities in Western China is closely related to economic development. The GDPP directly affects the economic and social benefits of resource-based cities. Restricted by geographical location, resource dependence, and policy support, the level of economic development of different resource-based cities in Western China is also different, which makes the static efficiency of green transformation different. Since 2010, China has gradually increased the undertaking of industries in Western China, implemented the green Belt and Road Initiative, and further promoted the strategy of western development in the 13^th^ Five-Year Plan. By increasing the proportion of the local transformation of resources and using industry to drive regional poverty alleviation, it has effectively promoted the rapid economic growth of resource-based cities in Western China, provided more material basis for the green transformation of resource-based cities as well as greatly improved static efficiency. In the early days of COVID-19, China's economy had a short shrinkage. But after the second quarter, with the effective control of COVID-19 in China and the continuous introduction of the “six stability” and “six guarantees” policy measures, China's economy began to steadily recover and return to the pre-epidemic level. Today, most cities in China, including resource-based cities in the western region, have realized continuous regional economic growth, providing strong material support for the improvement of static efficiency of green transformation.

Third, HC has negative effects on static efficiency. This may be due to the geographical location of western resource-based cities that is relatively remote and the economic development is relatively backward, resulting in a single level and low level of HC. Although the cities have adopted more talent introduction strategies, they still fail to attract more high-level talents. While resource-based cities lack core talents, homogenized and low-level human resources are relatively crowded. Due to the above reasons, the HC factor in the research range fails to play its endogenous dynamic role but hinders the improvement of static efficiency of green transformation of resource-based cities in Western China. This also points out that western resource-based cities should give more focus on the HC in the process of promoting green economic recovery and realizing green transformation in the post-epidemic era. The problems of education loss and population mobility caused by COVID-19 have further increased the pressure of HC to promote the static efficiency of resource-based cities' green transformation in western China.

Fourth, the coefficient of AIS, ER, and URB is positive and the coefficient of RO is negative, but they all fail to pass the significance test.

#### Influence of Different Factors on Dynamic Efficiency of Green Transformation

On the basis of testing the influence of different factors on the static efficiency of green transformation in resource-based cities in Western China, and in view that city green transformation is a long-term development process, we further investigate the impact of various factors on the intertemporal efficiency of green transformation from a dynamic perspective, and then the regression analysis of Model 2 is made correspondingly.

To obtain more accurate regression results and control the influence of the explained variable with one-period lag, this article adds the first-order lag term of the explained variable MI in the regression model as an instrumental variable, but this will inevitably lead to endogeneity problems. For dynamic panel data, deviations will be made in the estimator if panel data regression models, such as OLS, fixed effects, or random effects, are adopted. The system Gaussian Mixed Model (GMM) can effectively solve the potential endogeneity problem of model variables. Its basic method is to introduce the N-order lag term of the explained variables into the model as instrumental variables to alleviate the influence of endogeneity and ensure the effectiveness and consistency of estimators (40). Therefore, this article adopts the two-step system GMM method to estimate the model. At the same time, the Sargan test and Arellano Bond test are carried out on the basis of regression results. The Sargan test is used to judge whether the set tool variables are over-identified. The original hypothesis is “all the selected tool variables are valid.” If the *P* > 0.1, it means that the original hypothesis cannot be rejected, that is, the selected tool variables are valid. The Arellano Bond test is used to determine whether the error item has a sequence correlation problem. The *P*-value of AR (1) should be <0.1, that is, the original hypothesis of “no sequence autocorrelation” is rejected, indicating that the model has first-order autocorrelation. Meanwhile, the *P*-value of AR (2) should be > 0.1, which is, the original hypothesis of “no sequence autocorrelation” is accepted, indicating that the model has no second-order autocorrelation problem.

The test results show that the Sargan test result of model 2 is 1.000, far >0.1, so the original hypothesis is accepted, indicating that the instrumental variables selected in the estimation process are effective. The AR (1) *P* < 0.1, and the AR (2) *P* > 0.1, indicating that the model has first-order autocorrelation but no second-order autocorrelation. Therefore, the setting of the dynamic panel data model is reasonable.

Table 4 presents the specific regression results of dynamic efficiency. The results show that the regression coefficient of AIS is significantly positive, indicating that the advancement of industrial structure has significantly promoted the dynamic efficiency of green transformation in the western resource-based cities in a long term. This conclusion is consistent with the research results of scholar Zhao et al. (36). On one hand, this result is consistent with the general rule of industrial structure evolution. That is, the industrial structure of resource-based cities becomes more and more advanced through the increase of the proportion of tertiary industry and the decrease of the proportion of the secondary industry, which leads to the significant improvement of ecological and environmental problems. On the other hand, the characteristics of industrial structure determine the industrial distribution and development pattern of regional energy consumption and energy emission (41). With the introduction and implementation of a series of industrial support policies by the government in Western China in recent years, some highly polluting enterprises in resource-based cities have gradually withdrawn, while the number of knowledge-intensive and technology-intensive enterprises has increased. It increases the proportion of the tertiary industry, reduces energy consumption and pollutant emission, and promotes the green sustainable development of western resource-based cities. During the COVID-19 period, the tertiary industry was most directly affected by the epidemic. But thanks to China's great epidemic prevention measures, the cities all over the country returned to work and production in an orderly manner. In general, it has not affected the advancement of the western resource-based cities and even the whole country's industrial structure and the promotion of the 13th Five-Year plan. After entering the “14th five-year plan” period, the fluctuation of the output value proportion of the tertiary industry will be controlled within a reasonable range, and the development momentum of the emerging digital economy and service economy is good. If it continues to develop on this basis, the epidemic will not have a fundamental impact on the dynamic development process of green transformation, and the advancement of industrial structure will continue to promote the green transformation of resource-based cities in western China.

The correlation is positive between TI and the dynamic efficiency of green transformation in resource-based cities, which becomes significant at the statistical level of 5%. It indicates that TI can effectively reduce environmental pollution and improve sustainable development capacity, which is vital for the green transformation and development of resource-based cities in Western China (42, 43). In recent years, the state and the government of resource-based cities have continued to increase science and technology funds to support TI in the region. At the same time, governments have strengthened the green transformation of traditional industries, continuously improving equipment and tackling critical problems about core technologies on new energy and recyclable technologies. These have vigorously promoted the development of city environmental protection and further implemented the policies related to urban green environmental protection. To a certain extent, these help western resource-based cities achieve “overtaking on curves” in green transformation and sustainable development. COVID-19 is highly infectious. During the epidemic period, we should try to avoid contact with people. Under this control request, we will continue to innovate in the field of science and technology, represented by robots and artificial intelligence, and big data has been innovatively applied in epidemic prevention and control. Through the empowerment of science and technology and the replacement of labor with machines, the contact between people is reduced, the risk of epidemic spread is reduced, and the ecological and environmental pressure caused by human pollution is also reduced, which is conducive to the green transformation and development of resource-based cities in Western China.

Environmental regulation (ER) also has a positive impact on the dynamic efficiency of green transformation of resource-based cities in western China. It shows that the higher the level of ER, the better the development of city green transformation. In other words, long-term and reasonable ER can improve the dynamic efficiency of green transformation of resource-based cities. This may be because since the 11th and 12th Five-Year Plan, governments in Western China have gradually established a number of legal systems and policy guidance and unswervingly promoted green development and improved the environmental governance system. Taking the Environmental Protection Law of the People's Republic of China as the fundamental criterion for ecological and environmental protection in all parts of the west, different ERs and measures have been approved and issued in accordance with local conditions, such as Recent Soil Environmental Protection and Comprehensive Treatment Plan of Yunnan Province, the Environmental Protection Regulations of Inner Mongolia Autonomous Region, etc. Local governments also use their administrative power to strengthen environmental protection, including suspending high-pollution and high energy consumption plants, monitoring and controlling the source of industrial pollution, making marginal cost of pollutant companies close to marginal social cost. At the same time, the governments of resource-based cities in Western China have increased their investment in environmental pollution control. The multi-pronged approach has achieved remarkable results in pollution control and emission reduction, which effectively promotes the region's green transformation and development. Affected by the epidemic, the government has strengthened the environmental supervision of cities and enterprises to prevent the spread of the virus. This measure has accelerated the green transformation of the industry, promoted the green transformation of resource-based cities in western China and regional sustainable development.

The regression coefficient of GDPP is positive, indicating that under certain conditions, the higher the economic development level of resource-based cities in Western China is, the greater the positive impact it has on the dynamic efficiency of regional green transformation, and the more conscious and capable the region will be to promote the development of green transformation. In resource-based cities with a high GDPP, the government and the people tend to invest more material and HC. Through the development of high-tech R&D, equipment updating and innovation, and ecology of science and technology, the production efficiency can be improved while the emission reduction effect of science and technology can be brought into play to effectively reduce urban pollution. It also promotes green development which has low energy consumption, low pollution, high quality, and high efficiency. With the deepening of the concept of sustainable development and the One Belt and One Road initiative raised by general secretary Xi Jinping, the economy of the western resources-based city continues to improve. The cities take advantage of resources and energy to speed up the industrial transformation, build a sound environment to attract investment. The accumulation of financial strength for the transformation of green resources has fundamentally promoted the green sustainable development of resource-based cities. China is the first country to recover from COVID-19 in the world. The western resource-based cities, which is the same as the national economic development situation, have achieved economic recovery and sustained growth after a short period of shrinkage. This shows the economic resilience of resource-based cities in Western China and provides a strong endogenous impetus for the realization of green transformation.

The regression coefficient of URB on the dynamic efficiency of green transformation is positive, and it is significant at the 5% level. The research shows that the higher the URB, the stronger the promotion effect on the green transformation development of resource-based cities, which is consistent with the research results of Gao (44). A healthy ecological environment cannot be separated from the material basis and technological conditions. Urbanization, as a new driving force for economic development, forms an interdependent relationship with regional ecological environment protection (45, 46).

Through the eastern industrial transfer, trade exchanges, and cooperation with countries and regions along the Belt and Road, it has provided abundant employment opportunities, attracted a large number of rural population to the cities, and developed the urban economy. This has fundamentally promoted the urbanization process of the western resource-based cities. These cities have been rapidly facilitating the urbanization process, effectively gathering abundant capital and labor resources, introducing and developing advanced technology and equipment, and then they begin to be able to deal with various environmental pollution and other problems in a centralized manner. In addition, the human resources reserve in the urbanization construction also plays its due role, which helps to tap and release the green potential of the city, and promotes the green transformation and development of the western resource-based cities as a whole. Besides, the human resource in the urbanization construction also makes a contribution, which helps release the potential of cities, and promotes the green transformation development of resource-based cities in Western China as a whole. The outbreak of COVID-19 has increased the living pressure of residents in western resource-based cities. Especially during the closed management period, it is necessary to guarantee the basic living needs and the normal operation of the city while ensuring the safety of life. But at the same time, the epidemic has also strengthened the development of the urban medical and health industry and digital industry and deepened the city and its residents' awareness of the green industry and green life. Therefore, in the post-epidemic era, the improvement of URB can effectively promote the green transformation of resource-based cities in western China.

Both RO and HC have negative effects on dynamic efficiency, but they do not pass the significance test. However, it does not mean that the two have no impact on the green transformation development of resource-based cities. It may need to be estimated with data included in a longer time series in the future.

## Conclusion and Enlightenment

This article takes 38 resource-based cities in Western China from 2005 to 2016 as the research object, evaluates the static efficiency and dynamic efficiency of green transformation of each city by using the super-SBM model and the MI model of the DEA method and takes the two efficiency values as explained variables. We carry out Panel regression analysis by using the panel Tobit model with random effects and two-step system GMM method to comprehensively investigate the impact of different factors on the green transformation efficiency of resource-based cities in Western China. The main conclusions are as follows: first, the static efficiency of green transformation of resource-based cities in western China is generally low with uneven development. The regional green transformation needs to be further improved. Second, in the study area, the dynamic efficiency of green transformation of resource-based cities in western China shows an upward fluctuation and an accelerating trend. The overall development situation of green transformation is desirable. Third, the impact of TI and economic development level on the static efficiency of western resource-based cities' green transformation is significantly positive, while the impact of HC on static efficiency is significantly negative. It shows the necessity to strengthen the support for TI in the green transformation stage of western resource-based cities and improve the city's economic development. At the same time, the government needs to further explore the potential power of HC on the green transformation of resource-based cities. Fourth, the AIS, TI, ER, GDPP, and URB all have a significantly positive effect on the dynamic efficiency of green transformation, indicating that the improvement of the above factors will be conducive to the green transformation development of resource-based cities in western China in a long run.

Based on the special background of the global spread of COVID-19 and the above research conclusions, this article holds that, in the short term, COVID-19 is likely to remain, so the government should combine economic recovery with green transformation and integrate the concept of green sustainable development into all aspects of policy formulation, industrial layout, and production and life. It is expected that the resource-based cities in Western China will smoothly pass through the green transition window period and epidemic period while the economy continues to grow. In the medium and long term, we need to plan ahead and make a long-term layout, summarize the experience and lessons brought by the outbreak of COVID-19, improve the resilience of western resource-based cities to cope with sudden disasters and ecological changes, make innovation the first driving force and green the internal vitality, so as to contribute to China's sustainable development. This article draws the following enlightenment: first, although the efficiency level of green transformation of resource-based cities in western China has been improving year by year, it is still uneven and unable to promote the overall green sustainable development in the western region. The government of resource-based cities in Western China should focus more on the difference in green transformation efficiency among cities, formulate targeted policies according to local conditions, and realize the comprehensive, harmonious, and sustainable development of humans and nature in the region. 1) Local governments should continue to optimize the industrial structure and develop the tertiary industry. Although the outbreak of COVID-19 has brought unprecedented pressure to the tourism service industry of resource-based cities in western China, it has fully stimulated the development demand and potential of the urban digital economy. The traditional way of trade is under great pressure. The advantages of digital and accurate modern commercial trade are becoming increasingly obvious. E-commerce online shopping has become a new form of trade with its high efficiency and point-to-point characteristics, In addition, online industries, such as online entertainment, online conference, and office, are also growing rapidly. Therefore, resource-based cities in western China should seize the advantages of geographical location and opportunities of the times, actively participate in the construction of the new industrial chain of “One Belt and One Road,” cultivate and strengthen characteristic industries, develop emerging industries, improve the proportion of the tertiary industry, and promote the AIS. 2) The government should increase investment in science and technology and improve the level and intensity of technology innovation, especially the R&D and application of green technologies. On March 2, 2020, when General Secretary Xi Jinping inspected the COVID-19 prevention and control research work in Beijing, he stressed that “human development cannot overcome major disasters and epidemics without scientific development and TI” Western resource-based cities should seize the opportunity brought by the epidemic to promote social and TI, vigorously develop green technology, promote the construction of new infrastructure, and speed up the transformation of old and new driving forces. 3) The government should set up ER policies according to local conditions, improve decision-making level, avoid “one size fits all.” The impact of the epidemic poses a severe threat to the ER of resource-based cities in western China. Therefore, in the post-epidemic era, it is necessary to explore a differentiated ER model that takes into account both ecological civilization and economic and social development, improves the flexibility, pertinence, and effectiveness of ER, and grasps the ecological red line. All law enforcement departments should properly implement their responsibilities. The government should establish and improve the supervision and supervision mechanism for the development of green transformation, improve the supervision, increase the illegal cost of enterprises, establish and improve the compensation mechanism for ER, and formulate policies according to local conditions in western resource-based cities to improve the local ecological environment, so as to accelerate the green transformation in the post epidemic era. 4) The government should continue to push forward the city's economic development and improve urban economic resilience. In the post epidemic era, on the basis of fully doing a good job in epidemic prevention and control, the governments of resource-based cities in the West should have an overall awareness, deal with the relationship between the government and the market, the relationship between capital and labor, the relationship between supply and demand and the difference in the GDPP between regions, support the development of micro, small and medium enterprises, and provide them with financial, technical, policy and other assistance, guard against financial risks, and improve economic resilience. 5) The government should continue to facilitate the urbanization process, strengthen the construction of new urbanization, and improve the livable degree of cities by realizing city low-carbon production. In the post epidemic era, the government of resource-based cities in Western China should focus on the people, promote employment and protect people's livelihood, implement the employment priority policy, promote employment through multiple channels, give priority to ensuring the employment of low-income groups, provide training and services for laid-off reemployment, coordinate urban and rural development, and ensure the normal progress of urbanization in resource-based cities. 6) It is worth mentioning that HC was not found positive in increasing the green transformation efficiency in this study. So in the future, the government should improve the talent introduction system to attract medium and high-end talents, adjust the current utilization mode of HC to fully tap their potential power, make HC play a full role in the post epidemic era, and drive resource-based cities in the west to realize green economic recovery.

Second, with the experience of green transformation of resource-based cities in the central and eastern regions, such as Xuzhou and Zaozhuang, western cities should continue to support their dominant industries while expanding new economic growth points and accelerating the construction of public health, disaster prevention, and mitigation, comprehensive transportation and other projects to improve the green transformation efficiency in the post epidemic era.

Finally, western resource-based cities should improve green production capacity and resource utilization efficiency to reduce resource investment and waste, accelerating the green transformation process of resource-based cities in western China, realizing the win-win situation of “mountains of gold and silver” and “clear waters and green mountains” in the near future, and contributing China's strength to the world's green recovery and sustainable development in the post-epidemic period.

## Discussion

At present, resource-based cities in western China are in the window period of green transformation, the epidemic prevention and control period, and the construction period of the new development pattern of the national “double-cycle,” which is also a historical-critical period for the economic and social development of the western region and China. While keeping up with the characteristics of the times, this article uses the efficiency characteristics to investigate the green transformation achievements of western resource-based cities from static and dynamic perspectives, and regresses the key influencing factors, so as to provide a more targeted and enlightening basis for the improvement and development direction of green recovery and sustainable development in the post epidemic era. Therefore, compared with the existing literature, on the one hand, the perspective of this study is more comprehensive, scientific, and directional. On the other hand, this study further enriches the regional research system of green transformation of resource-based cities in China. In addition, in reality, the factors affecting the green transformation of resource-based cities in western China will be more complex. Therefore, it will be the focus of future research to continuously track and introduce new explanatory variables and conduct more microscopic studies based on different types of resource-based cities.

## Data Availability Statement

The original contributions presented in the study are included in the article/supplementary material, further inquiries can be directed to the corresponding author.

## Author Contributions

JZ contributed to the motivation, the interpretation of the methods, the data analysis and results, and provided the draft versions and revised versions. SW provided the data results, the revised versions, and references. All authors contributed to the article and approved the submitted version.

## Funding

This research was funded by Research on Dual Incentive Model of Carbon Reduction and Emission Reduction Target, Government Competition and Urban Environmental Governance, which was the research and planning fund for humanities and social sciences of MOE (Ministry of Education in China). The grant number is 21YJA790086.

## Conflict of Interest

The authors declare that the research was conducted in the absence of any commercial or financial relationships that could be construed as a potential conflict of interest.

## Publisher's Note

All claims expressed in this article are solely those of the authors and do not necessarily represent those of their affiliated organizations, or those of the publisher, the editors and the reviewers. Any product that may be evaluated in this article, or claim that may be made by its manufacturer, is not guaranteed or endorsed by the publisher.
